# Asymptomatic or symptomatic SARS-CoV-2 infection plus vaccination confers increased adaptive immunity to variants of concern

**DOI:** 10.1016/j.isci.2022.105202

**Published:** 2022-09-23

**Authors:** Peifang Sun, Irene Ramos, Camila H. Coelho, Alba Grifoni, Corey A. Balinsky, Sindhu Vangeti, Alison Tarke, Nathaniel I. Bloom, Vihasi Jani, Silvia J. Jakubski, David A. Boulifard, Elizabeth Cooper, Carl W. Goforth, Jan Marayag, Amethyst Marrone, Edgar Nunez, Lindsey White, Chad K. Porter, Victor A. Sugiharto, Megan Schilling, Avinash S. Mahajan, Charmagne Beckett, Alessandro Sette, Stuart C. Sealfon, Shane Crotty, Andrew G. Letizia

**Affiliations:** 1 Naval Medical Research Center, Silver Spring, MD, USA; 2 Icahn School of Medicine at Mount Sinai, New York, NY, USA; 3 Precision Immunology Institute, Icahn School of Medicine at Mount Sinai, New York, NY, USA; 4 La Jolla Institute for Immunology, La Jolla, CA, USA; 5 Henry M Jackson Foundation for the Advancement of Military Medicine, Inc., Bethesda, MD, USA; 6 Stockholm University, Stockholm, Sweden; 7 Navy Medicine Readiness and Training Command, Jacksonville, FL, USA; 8 Department of Medicine, University of California San Diego, San Diego, CA, USA; 9 Naval Medical Research Unit-2-Asia, Singapore

**Keywords:** Biological sciences, immunology, microbiology

## Abstract

The ongoing evolution of SARS-CoV-2 requires monitoring the capability of immune responses to cross-recognize Variants of Concern (VOC). In this cross-sectional study, we examined serological and cell-mediated immune memory to SARS-CoV-2 variants, including Omicron, among a cohort of 18-21-year-old Marines with a history of either asymptomatic or mild SARS-CoV-2 infection 6 to 14 months earlier. Among the 210 participants in the study, 169 were unvaccinated while 41 received 2 doses of mRNA-based COVID-19 vaccines. Vaccination of previously infected participants strongly boosted neutralizing and binding activity and memory B and T cell responses including the recognition of Omicron, compared to infected but unvaccinated participants. Additionally, no measurable differences were observed in immune memory in healthy young adults with previous symptomatic or asymptomatic infections, for ancestral or variant strains. These results provide mechanistic immunological insights into population-based differences observed in immunity against Omicron and other variants among individuals with different clinical histories.

## Highlights

• Adaptive immune response is similar after symptomatic or asymptomatic infection
• Vaccination after SARS-CoV-2 infection strongly boosted adaptive immunity
• Memory B and T cell responses to Omicron were greater among vaccinated participants

## Graphical abstract

**Figure:**
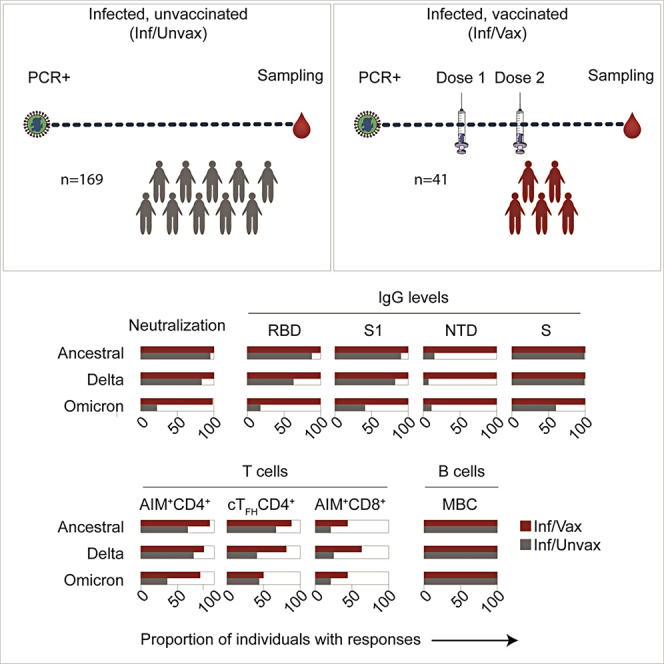

## Introduction

The SARS-CoV-2 Omicron variant (B.1.1.529) was classified as a Variant of Concern (VOC) in November 2021 by the World Health Organization (WHO) and the United States Centers for Disease Control and Prevention (CDC), and became the dominant variant in the US and globally (CDC, 2022) shortly thereafter.

Compared to the ancestral Wuhan-Hu-1 strain, Omicron has 37 mutations in the S protein (Cameroni et al., 2022; McCallum et al., 2022). Fifteen of these mutations occur in the receptor-binding domain (RBD); of these, 10 are located at the ACE2-RBD interface (Yin et al., 2022). Another 11 mutations are located in the N-terminal domain (NTD) (McCallum et al., 2022). The S protein mutations within the Omicron variant modulate the receptor-binding affinity, and contribute to the evasion of immune protection derived from infection and/or vaccination, and impair monoclonal antibody-based immunotherapy (Cameroni et al., 2022; Cao et al., 2022; Carreno et al., 2022; Cele et al., 2022; Lupala et al., 2022; Mannar et al., 2022). As of Nov 2021, approximately 80% of the US population had received vaccines designed based on the Wuhan-Hu-1 strain and approximately 30% of the global population had experienced infection with the Ancestral strain or other variants circulating prior to Omicron (Collaborators 2022; Kleynhans et al., 2022).

Young adults play an important role in sustaining resurgent SARS-CoV-2 transmission (Monod et al., 2021). Comprehensive assessments of adaptive immunity including cross-reactivity to Omicron after infection with Ancestral strain SARS-CoV-2, or vaccination, or a combination of both in the general population—and in young adults in particular—can inform public health policies including vaccination strategies.

Infection can elicit durable memory B and T cell responses with similar or lower magnitudes than that of vaccine-induced immunity (Dan et al., 2021; Greaney et al., 2021; Marcotte et al., 2022; Naranbhai et al., 2022; Zhang et al., 2022). A combination of infection and vaccination, known as hybrid immunity (Goldblatt 2022), outperforms the immunity induced by infection or vaccination alone (Abu-Raddad et al., 2021; Lumley et al., 2021). The synergistic effect between infection and vaccination is in part explained by substantial improvement in neutralizing antibody titers and breadth associated with the frequency and quality of memory B cells after infection (Crotty 2021; Goel et al., 2021; Reynolds et al., 2021; Wang et al., 2021).

Additionally, the importance of T cells is evident through a correlation with the less severe disease after infection (Moderbacher et al. Cell 2020, Tan et al. Cell rep. 2020) and the onset of vaccine protection correlating with the appearance of T cell responses (Kalimuddin et al. Cell Rep 2021). Unlike serum antibodies, T cell responses induced by the Ancestral strain show high cross-reactivity to VOCs including Omicron (Gao, Cai et al., 2022; GeurtsvanKessel et al., 2022; Keeton et al., 2022; Liu et al., 2022; Tarke et al., 2022). The quantity and functional capabilities of T cells increase in individuals after infection plus vaccination as compared to those who were vaccinated but never infected (Bertoletti et al., 2021; Crotty 2021). Although these lines of evidence suggest that symptomatic infection followed by vaccination provides substantially more potent immune responses than either infection or vaccination alone, whether asymptomatic infection plus vaccination confers similar immunological benefits is less well established (Reynolds et al., 2020; Sekine et al., 2020; Boyton and Altmann 2021; Le Bert, Clapham et al., 2021), particularly in subpopulations such as young adults. Key questions, including the magnitude and longevity of hybrid immunity in these individuals and cross-reactivity to VOCs such as Omicron, remain poorly resolved, owing to challenges in identifying asymptomatic infections and differences in vaccination uptake between age groups.

This cross-sectional study characterized a cohort of previously SARS-CoV-2 infected United States Marines aged 18-21, some of whom remained unvaccinated and others subsequently received vaccination with two doses of an mRNA vaccine. Serum antibodies, memory B cells, memory CD4 T cells, and CD8 T cells were all characterized in individuals with asymptomatic or symptomatic infections followed by vaccination or no vaccination to determine the impact of symptomatology on immunological memory in this cohort of young adults.

## Results

### Cohort description

**Figure 1:**
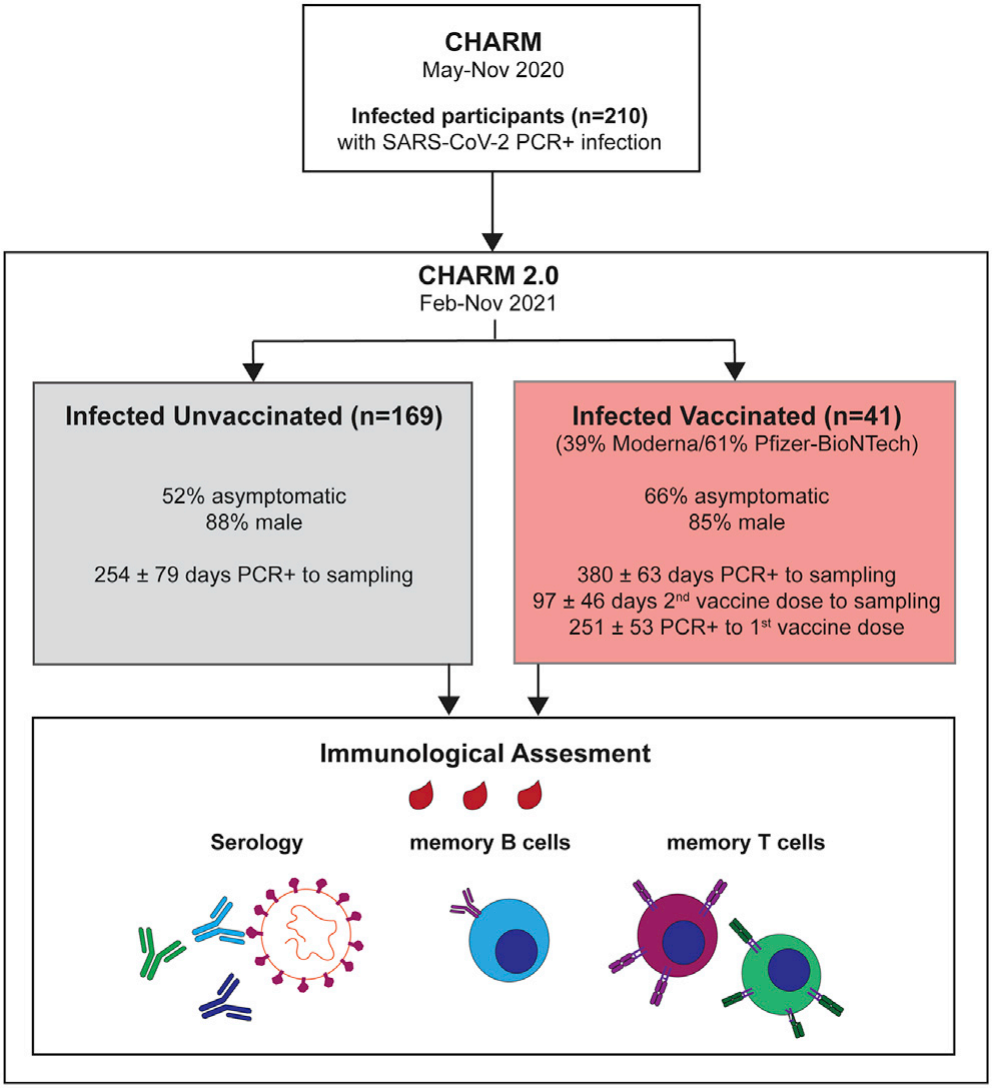
Study design and cohort description

The COVID-19 Health Action Response for Marines (CHARM) prospective cohort study was described previously (Letizia et al., 2020; Letizia et al., 2021b). CHARM 2.0 is a continuation of the CHARM study, in which participants enrolled between May and November 2020 were followed-up in a cross-sectional fashion. In this study, we included participants who had at least one PCR confirmation of SARS-CoV-2 infection during CHARM, and then provided at least one follow-up sample during CHARM 2.0. Overall, a total of 210 participants were included in the study, of whom 169 were infected and unvaccinated (80.5%) and 41 were infected and vaccinated with mRNA vaccines, with a second dose at least 28 days prior to their follow-up visit (29.5%, Figure 1), but had not received a booster vaccine.

The average age of the sample population at enrollment in the CHARM study was 19.1 ± 1.9 years. The participants self-identified their race as either White (68.6%), Black (13.8%), Asian (2.9%), American Indian/Alaska Native (1.4%), Hawaiian/Other Pacific Islands (0.5%) or non-specified (4.3%), and their ethnicity as Hispanic (29.0%), Non-Hispanic (45.2%) or non-specified (25.7%). A total of 86.7% of the participants in the study were male (87.6% in unvaccinated and 85.4% in vaccinated groups). The epidemiological characteristics of the SARS-CoV-2 outbreaks in CHARM were described previously (Lizewski et al., 2022). Five main phylogenetic virus clusters were identified, and all had sequences related to the Ancestral strain with the D614G amino acid mutation. Therefore, the documented exposure to SARS-CoV-2 virus of these participants is not associated with any of the VOCs (Letizia et al., 2021a). All participants who experienced symptoms were treated as outpatients and none required hospitalization (Letizia et al., 2020).

The average time interval between the first PCR confirmation in CHARM and the sample collection at CHARM 2.0 follow-up visits was 254 ± 79 days (ranging from 129 to 503 days) for the unvaccinated group. For the vaccinated group, the average time between the first PCR confirmation to the first dose of vaccine was 251 ± 54 days (ranging from 156 to 430 days). The average time between the second vaccine dose and sample collection was 97 ± 46 days (ranging from 34 to 189 days). Among the vaccinated participants, 39% had received the Moderna vaccine and 61% the Pfizer-BioNTech vaccine. Vaccine status and dates were confirmed using the participant’s Department of Defense (DoD) electronic medical record. The participants were divided into two groups based on their vaccination status and classified as infected-unvaccinated and infected-vaccinated hereafter. Of the 169 infected-unvaccinated participants 88 (52%) were asymptomatic and 81 (48%) symptomatic, while of the 41 infected-vaccinated participants 27 (66%) were asymptomatic and 14 (34%) symptomatic.

### mRNA vaccination enhances antibody response against omicron after asymptomatic or symptomatic infection

**Figure 2:**
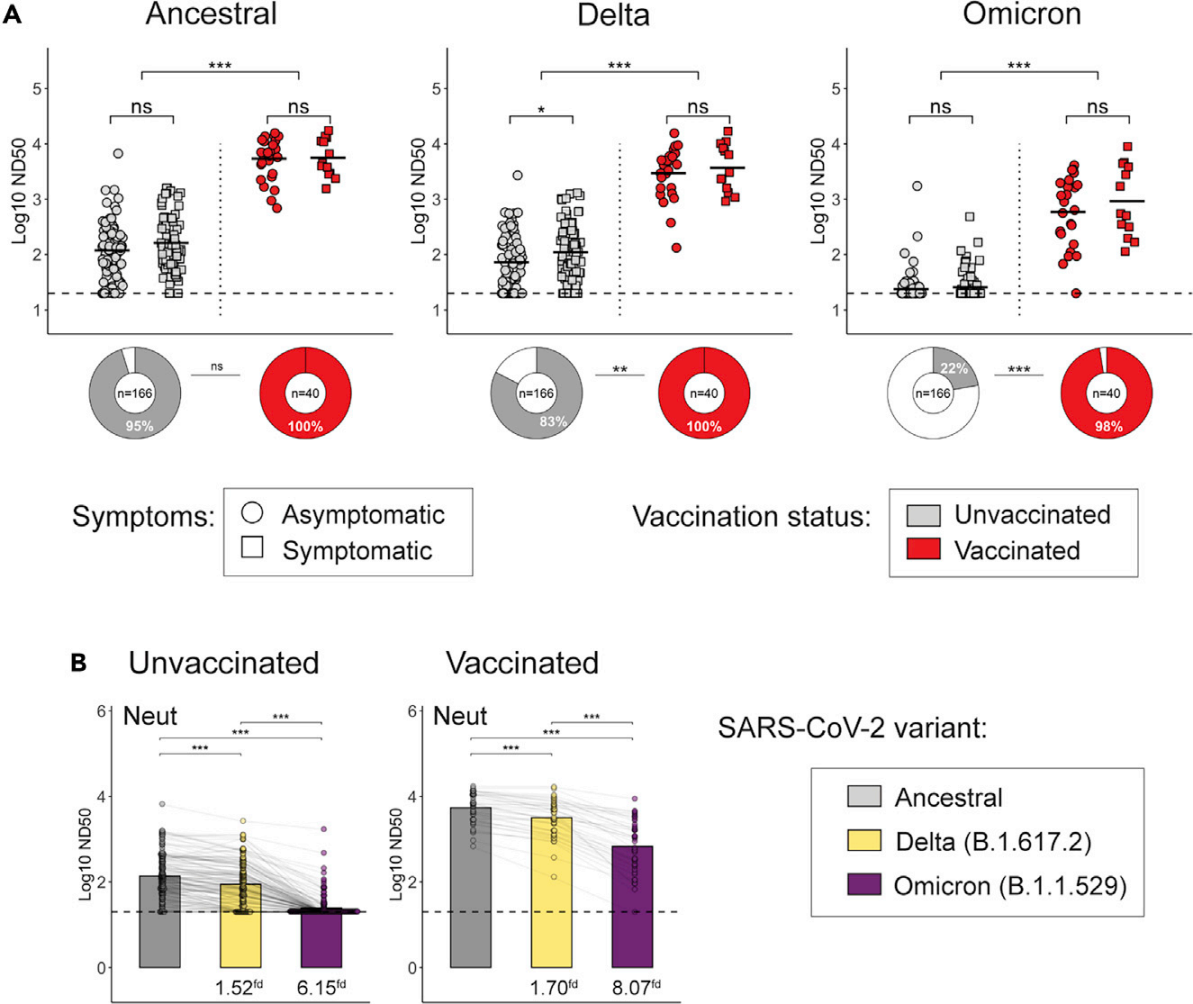
Vaccination enhances neutralizing antibody function against Delta and Omicron variants (A) ND_50_ in vaccinated and unvaccinated participants, stratified by the presence of symptoms. The frequency of positivity (ND_50_>20) between unvaccinated and vaccinated participants (doughnut plots) was compared using the Fisher Exact test (B) Comparison of neutralizing activity against Ancestral strain, Delta, and Omicron variants. Median fold-change decrease (^fd^) as compared to the Ancestral strain is indicated under Delta and Omicron bars and was calculated considering only samples with detectable neutralizing activity against the Ancestral strain (>20 ND_50_). Mean values are shown in (A) (black line) and (B) (bars). ND_50_ values were compared using the Mann-Whitney test. ∗∗∗p < 0.001; ∗∗p < 0.01; ∗p < 0.05; ^ns^p ≥ 0.05.

We evaluated the neutralization titers of sera from the infected-unvaccinated and infected-vaccinated groups to the Ancestral strain (with the D614G change), the Delta variant, and the Omicron variant using an established PV assay. In the unvaccinated group, a high percentage of participants had detectable neutralizing activity against the Ancestral strain and the Delta variant (95.2% and 82.5%, respectively, Figure 2A). Only 22.3% of the participants in this group showed any detectable neutralization activity against the Omicron variant.

In striking contrast to the unvaccinated group, virtually all infected-vaccinated participants had detectable neutralizing activity to the Ancestral (positivity rate: 100%), Delta (positivity rate: 100%), and Omicron variants (positivity rate: 98%) (Figure 2A). In addition, vaccinated participants showed significantly enhanced ND_50_ levels against all three strains (Ancestral: median 6330, 95% confidence interval [CI] 4245-9528; Delta: median 3726, CI 2352-5811; Omicron: 764, CI 337-1639, p < 0.001), when compared to unvaccinated participants (Ancestral: median 129, CI 101-149, p < 0.001; Delta: median 77, CI 67-109, p < 0.001; Omicron: median 20, CI 20-20, p < 0.001).

Both unvaccinated and vaccinated groups showed a pronounced fold-decrease (calculated including only samples with detectable ND_50_ to Ancestral strain) in ND_50_ titers against Omicron (median fold decrease 6.15 and 8.07, respectively) and Delta (1.52 and 1.70, respectively) in comparison to the Ancestral strain (Figure 2B). Of note, as the majority of the unvaccinated participants had undetectable neutralization antibody against Omicron, the median fold decrease calculation in this group is likely an underestimate. Neutralization activity was similar regardless of symptoms except for higher neutralizing titers to the Delta variant in symptomatic unvaccinated participants compared to asymptomatic unvaccinated participants (p = 0.049) (Figure 2A).

**Figure 3:**
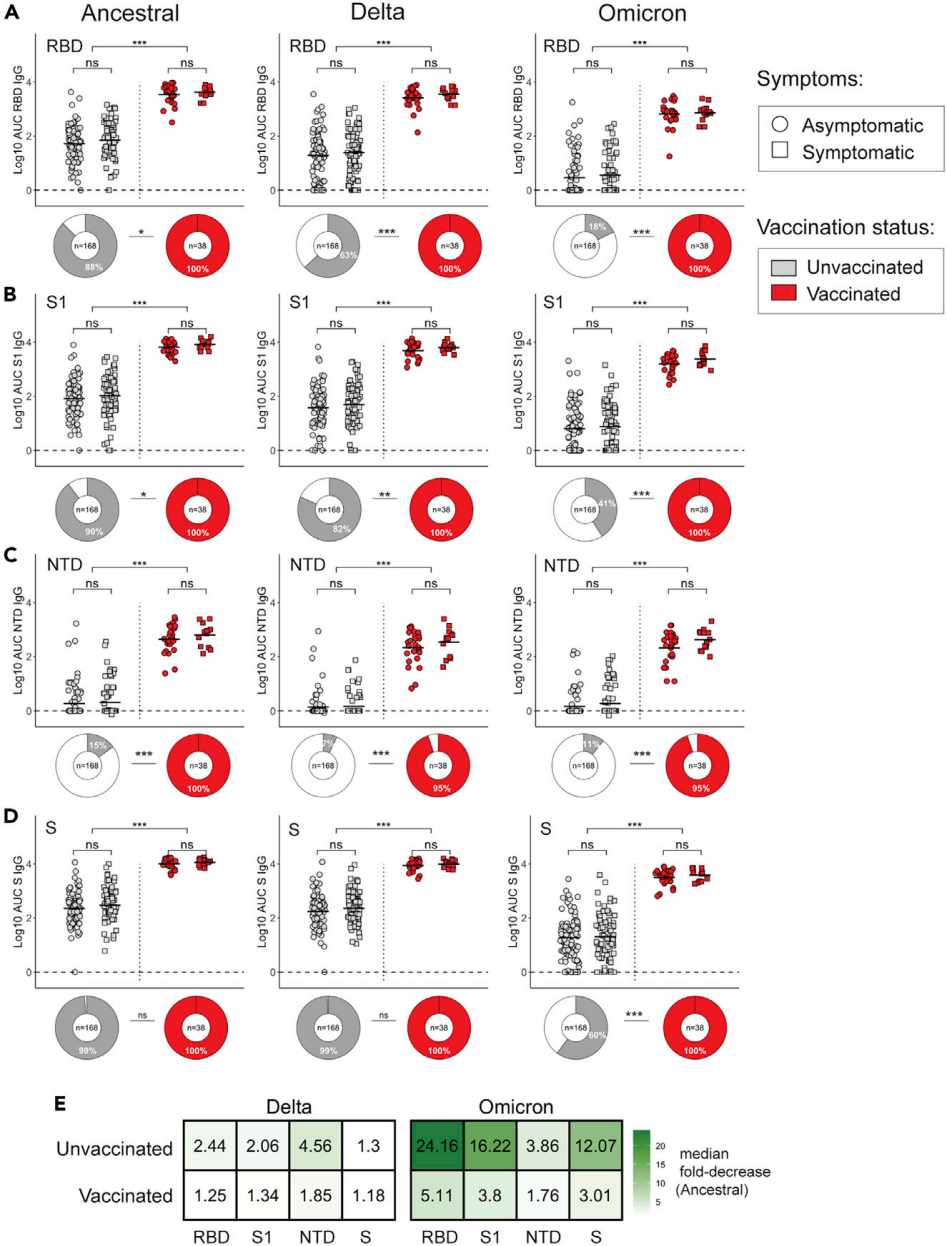
Vaccination enhances serum IgG binding SARS-CoV-2 S protein from ancestral strains and variants of concern (A–D) Percentage of samples with a titer of at least 150 for each antigen is indicated in the doughnut plots (A–D). Area Under the Curve (AUC) for IgG binding to RBD, S1, S (trimer), and NTD are shown in (A–D), respectively. AUC were compared using the Mann-Whitney test, and frequency of positivity between unvaccinated and vaccinated participants (doughnut plots) was compared using the Fisher Exact Test (∗∗∗p < 0.001; ∗∗p < 0.01; ∗p < 0.05; ^ns^p≥0.05. Mean values are shown in (A–D) (black lines). (E) Heatmap indicates the median fold-change decrease as compared to the Ancestral strain for Delta and Omicron variants, considering only samples with IgG titers (≥150) for the Ancestral strain.

Next, we evaluated the ability of serum antibodies to bind different regions of the S protein of the Ancestral and the variant strains. Similar response patterns were seen for neutralization activity and IgG binding activity in RBD, S1, and NTD domains, as well as in S trimers. Significantly higher (p < 0.001) IgG binding AUC for all antigens of the three strains were recorded for the vaccinated group compared to the unvaccinated group, both in terms of positivity rate (IgG titers ≥150) and response magnitude (Figure 3).

For the unvaccinated group, the IgG positivity rates for RBD, S1, and S of the Ancestral strain were 87.5%, 89.9%, and 98.8%, respectively; for Delta were 63.1%, 81.5% and 99.4%, respectively; but for Omicron were only 17.2%, 41.1%, and 60.1%, respectively (Figure 3, doughnut plots). In contrast, virtually all vaccinated participants showed positive IgG titers against all antigens tested for all strains, including Omicron.

The low antibody binding against Omicron was also reflected quantitatively by fold decreases in AUC relative to the Ancestral strain (Figures 3 and S1). The fold decreases (calculated including only samples with IgG titers to Ancestral strain ≥150) in IgG binding to Omicron RBD, S1, NTD, and S were greater in unvaccinated participants (24.16, 16.22, 3.86, 12.07, respectively) than in vaccinated participants (5.11, 3.8, 1.76, 3.01, respectively) (Figures 3E and S1).

There were no significant differences between the asymptomatic and symptomatic subgroups within each of the vaccinated and unvaccinated groups in IgG antibody binding to any antigens and variants (Figures 3A–3D).

We next investigated if the time between infection and vaccination, which ranged from 156 to 430 days, had an effect in the magnitude of the serum antibody levels. Using linear regression models, we found a trend towards higher ND_50_ levels in participants with longer times between the first PCR + and the first dose of the vaccine (Figure S2A). However, this upward trend was significant only for neutralizing activity against Omicron (p-values = 0.0851, 0.0793, and 0.0255 for Ancestral, Delta, and Omicron, respectively). We did not find a significant change in IgG binding to RBD, S1, NTD, or S of any of the variants based on the time between infection and vaccination (Figures S2B–S2E).

Overall, antibody responses to the Ancestral strain were still detected in infected participants about 6-14 months post-infection. However, the neutralizing and binding activity to RBD, S1, and S of Omicron was markedly decreased, as compared to the Ancestral reactivity. Vaccination greatly improved antibody responses to all antigens for all strains. No significant differences were noted in antibody binding and neutralization responses between the asymptomatic or symptomatic infection groups.

### Immunization increases the frequency of S-binding memory B cells in convalescent subjects

**Figure 4:**
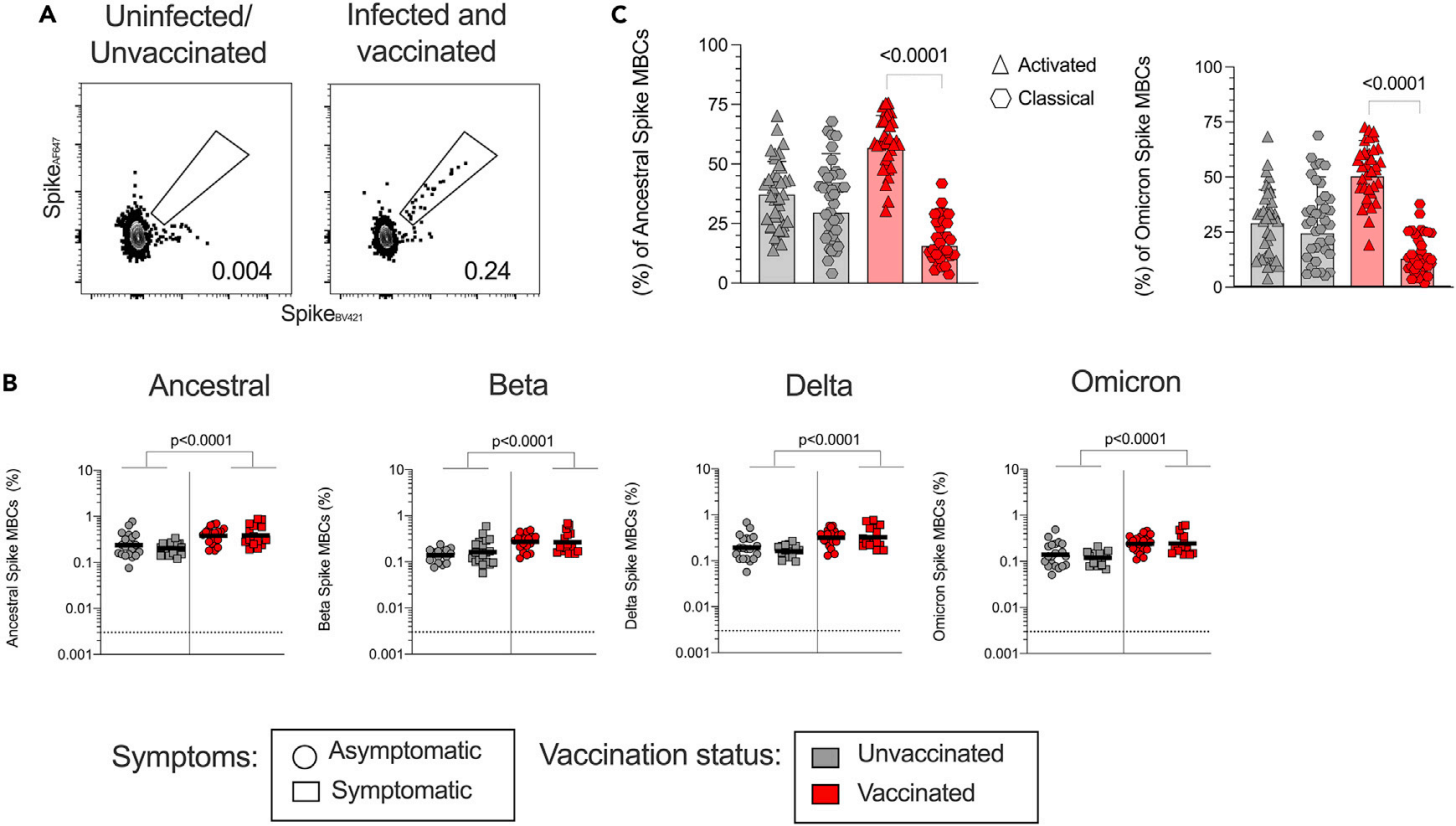
S-specific memory B cell response against Ancestral, Beta, Delta, and Omicron variants in COVID-19 convalescent subjects receiving mRNA immunization (A) Representative gating to detect S-binding MBCs. (B) Frequency of S-binding MBCs recognizing each of the variants and Ancestral. (C) Frequency of Classical (CD27^+^ CD21^+^) and Activated (CD27^+^ CD21^−^) S-binding MBCs recognizing Ancestral and Omicron. Statistics were performed using the Mann-Whitney test. Geometric mean (black lines panel B) with geometric SD is shown (panel C).

We assessed the frequency of S-specific Ancestral, Beta, Delta, and Omicron memory B cells using biotin-conjugated probes complexed with streptavidin (Figure 4A). The flow cytometry gating strategy is depicted in Figure S3. All participants had memory B cell responses to S protein above the limit of detection (100% positivity rate). The Ancestral and Omicron S-specific memory B cells in infected participants were on average 0.25% and 0.15%, respectively, of total memory B cells. For Beta and Delta, this frequency was 0.18% and 0.21%, respectively. In the vaccinated group, the frequency increased to 0.42% for Ancestral and 0.27% for Omicron (p < 0.0001). The frequency of Beta and Delta-reactive memory B cells also increased significantly after vaccination (0.30% and 0.36%, p < 0.0001). No difference in the frequency of memory B cells was observed between symptomatic and asymptomatic infection groups who remained unvaccinated (Figure 4B), or between symptomatic and asymptomatic infection groups who were vaccinated (Figure 4B). The frequency of memory B cells recognizing Beta and Omicron S was decreased, compared to the Ancestral strain, among both unvaccinated (Beta p = 0.0086, Omicron p < 0.0001) and vaccinated participants (Beta p = 0.0348, Omicron p = 0.0014) (Figures S4A and S4B).

Activated memory B cells (CD21^−^CD27^+^) were increased in the vaccinated group when compared to classical memory B cells (CD21^+^CD27^+^) (p < 0.0001), Figures 4C and S4C). The isotype of S-specific memory B cells was predominantly IgG (67.4%) followed by IgM (15.3%) and IgA (3.7%) in the unvaccinated participants. IgA^+^ memory B cell frequencies were low but significantly higher in unvaccinated participants compared to vaccinated participants (3.7% unvaccinated vs 1.8% vaccinated p = 0.0007, Ancestral S-binding memory B cells, Figure S4D). No differences in IgM S-specific B cells were detected between the groups (Figure S4D). Overall, infection followed by vaccination was associated with substantially increased memory B cell frequencies against S compared to infection alone.

### Higher S-specific T cell responses are induced by infection followed by vaccination

**Figure 5:**
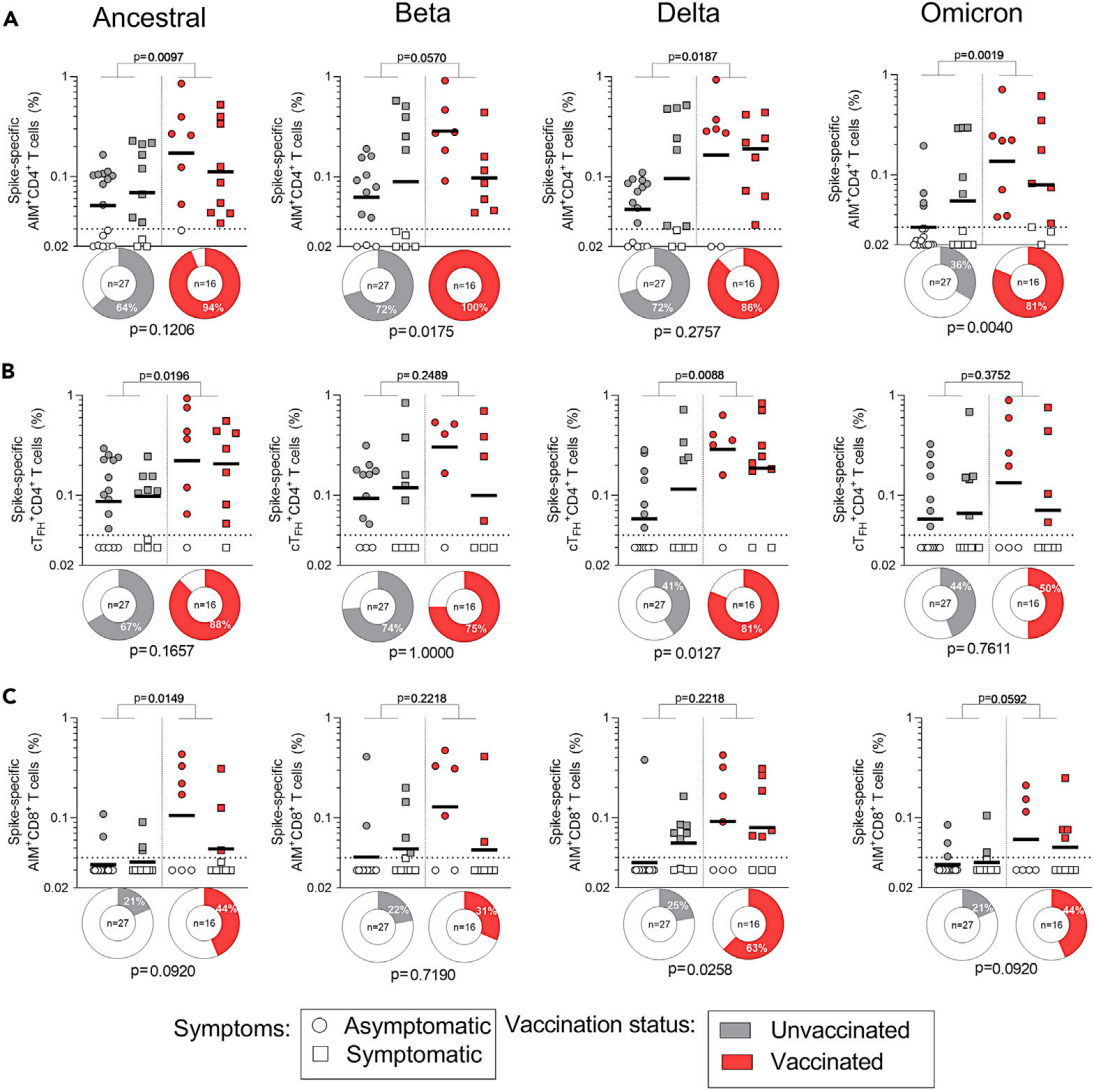
S-specific T cell response against Ancestral, Beta, Delta, and Omicron variants (A–C) Naturally infected unvaccinated (grey) and vaccinated (red) samples are analyzed in terms of the magnitude of response as the percentage of S-specific cells and as pie chart showing the frequency of responders for AIM^+^ (OX40^+^CD137^+^) CD4^+^ T cells (A), AIM^+^ (OX40^+^CD40L^+^) cT_fh_ cells (B) and AIM^+^ (CD69^+^CD137^+^) CD8^+^ T cells (C). In each cohort the negative samples are shown in white, circles represent asymptomatic infections while squares symptomatic infections and for both Geometric mean is shown (black lines). Comparison of the two cohorts in terms of magnitude and frequency of responses are performed by Mann-Whitney and Fisher Exact test, respectively.

We next characterized T cell responses to the Ancestral sequence and the panel of variants considered above. T cell responses were measured using an activation-induced marker (AIM) assay and the fraction of antigen-specific responses was defined by the expression of OX40^+^CD137^+^, and CD69^+^CD137^+^ for CD4^+^ T cells and CD8^+^ T cells, respectively (gating strategy shown in Figure S5), as previously reported (Tarke et al., 2022). SARS-CoV-2-specific memory cT_FH_ CD4^+^ T cells (CXCR5^+^) were quantified as OX40^+^CD40L^+^ cells. For these analyses, a total of 43 samples were used, 27 from unvaccinated and 16 from vaccinated participants (Figures 5 and S6). We measured memory recall responses by stimulating the cells for 24 h with peptide pools spanning the entire S protein and corresponding to the Ancestral sequence and each of the VOCs in the analysis.

A comparison between the vaccinated and unvaccinated groups identified significantly higher magnitude of CD4^+^ T cell responses for Ancestral (p = 0.0097), Delta (p = 0.0187) and Omicron (p = 0.0019) variants and a trend for higher responses in the context of the Beta variant (p = 0.0570). Similarly, at the level of frequency of positive responses (response rate, Figure 5A inset), we observed a significantly higher response among the vaccinated as compared to unvaccinated participants for Beta (p = 0.0175) and Omicron (p = 0.0040). Overall, the response rates were higher across different variants in the infected-vaccinated participants (range 81-100%) than among the infected-unvaccinated participants (ranged 36-72%) (p = 0.0219 by the Mann-Whitney test). The CD4^+^ T cells’ capability to recognize VOCs between the two groups was comparable (unvaccinated: p = 0.0835, vaccinated: p = 0.4607 by the Kruskal-Wallis test; Figure S6A).

S-specific cT_FH_ response rates were similar in the vaccinated group compared to the unvaccinated group across the various variants (unvaccinated range: 41-74%; vaccinated range 50-88%; p = 0.1954 by Mann-Whitney). However, the magnitude of S-specific cT_FH_ cell responses was significantly increased in the vaccinated versus unvaccinated groups in most comparisons (Ancestral p = 0.0196; Beta p = 0.2489; Delta p = 0.0088; Omicron p = 0.3752, by Mann-Whitney) (Figure 5B).

Finally, the CD8^+^ T cells responses rates were increased in the vaccinated group compared to the unvaccinated group (unvaccinated range: 21-25%; vaccinated range 31-63%; p = 0.0129 by Mann-Whitney). Significantly higher frequencies of Ancestral S-specific CD8 T cells were observed in the vaccinated group compared to the unvaccinated group (Ancestral p = 0.0149, Figure 5C). Overall, CD8^+^ T cells cross-recognized multiple variants in both unvaccinated and vaccinated groups (unvaccinated: p = 0.5038 vaccinated: p = 0.6723 by Kruskal-Wallis; Figure S6C). No differences were seen between the symptomatic and asymptomatic infection subgroups within infected-unvaccinated and infected-vaccinated groups for all T cell subsets analyzed and for all variants.

Overall, the results demonstrate that vaccination after infection induced higher T cell responses compared to infection alone. Furthermore, a prior asymptomatic infection had a comparable impact on the frequency of CD4^+^ T cell, cT_FH_ cell, and CD8^+^ T cell memory following vaccination compared to the enhancement seen with prior symptomatic infection.

## Discussion

We observed a similar adaptive immune response to the Ancestral strain and to VOCs in participants with mildly symptomatic infections as compared to asymptomatic infections either after infection alone or infection followed by mRNA vaccination. Our data solidifies the finding that asymptomatic infections can induce durable antibody responses and memory B and T cells just as well as mildly symptomatic infections (Abayasingam et al., 2021; Vo et al., 2022) and immunity can be boosted following vaccination. Since late summer 2020, adults between 20 and 49 years of age have become an important age group in terms of sustaining SARS-CoV-2 transmission (Monod et al., 2021). Data from our cohort, composed entirely of young, healthy adults who often experience asymptomatic infection, adds to our understanding of adaptive immunity among a group whose immune status is key to preventing onward transmission to more vulnerable populations.

Multiple studies comparing the immune response induced by infection-only and vaccine-only, which have been recently included in a comprehensive comparative study (Townsend et al., 2022), indicate that mRNA vaccine regimens induced more sustained antibody responses than infection. Other studies have compared infection-only, double dose-vaccine-only, booster vaccine doses, and/or infection plus vaccination (Carreno et al., 2022; Pajon et al., 2022; Lusvarghi et al., 2022) and showed that infection-only and two vaccine doses without prior infection provided no or poor neutralization to the Omicron variant. Sustained neutralization to Omicron required three exposures to the Spike protein either with three doses of vaccine or by combining infection with vaccination. Therefore, although these studies and ours differ in the timing and participant demographics, our results revealing the waning of immune responses post-infection was in line with these reports and emphasized the necessity of double doses of vaccination in the infected population to sustain a robust immune response to the Omicron variant.

We assessed the impact of infection symptomatology on vaccine immune responses, in conjugation with the assessment of the recognition of Omicron. Antibody neutralization and RBD-binding antibodies to Omicron were only detected in 22% and 18% of the unvaccinated participants. Impressively, after vaccination, ND_50_ and IgG to RBD of Omicron were both restored in nearly 100% of the participants. Infection followed by vaccination can improve the potency and breadth of the serological responses (Stamatatos et al., 2021; Turner, O'Halloran et al., 2021; Edara et al., 2022; Garcia-Beltran et al., 2022), as well as the breadth of the B cell memory response against VOCs (Wang et al., 2021). This is consistent with our data and suggests a similar immune response among young adults who experienced asymptomatic or mildly symptomatic infection and were subsequently vaccinated.

S-specific memory B cells were sustained in all participants to the Ancestral strain as well as to Delta and Omicron variants. Immune escape is evident in memory B cells recognizing Omicron, similar to previous reports (Tarke et al., 2022). Of note, a total of 40% of our unvaccinated study participants lacked binding antibodies to the trimeric S protein of Omicron. Some of these participants were simultaneously tested for memory B cells and showed detectable levels of Omicron S-reactive memory B cells. The data highlight that memory B cells are an important component of hybrid immunity recognizing VOCs, including Omicron. Serological immune results alone may not accurately reflect an individual’s immunity in terms of their ability to recognize VOCs upon infection.

Our study shows that vaccination after infection results in a higher T cell response compared to infection alone both in response magnitudes and frequencies. This phenomenon is most prominent on the S-specific CD4^+^T as compared to cTfh and CD8+T cells. In general, T cells responses are relatively well-preserved against SARS-CoV-2 variants in vaccination and infection (Gao et al., 2022; GeurtsvanKessel et al., 2022; Keeton et al., 2022; Liu et al., 2022; Tarke et al., 2022). Our study showed that regardless of vaccination status, when a positive T cell response was detected, it was able to cross-recognize multiple variants including Omicron, which is consistent with what was reported previously in the context of infection or vaccination without infection (Keeton et al., 2022; Liu et al., 2022; Tarke et al., 2022). Although a measurable T cell response to VOCs occurs in young adults after infection alone regardless of symptomatology, a greater response occurs after infection followed by vaccination.

Notably, one population-based vaccine/epidemiology study indicated that the interval between a prior infection and the first dose of vaccination is important, as an interval of 6 months or greater can significantly lower the risk of breakthrough infection (Abu-Raddad et al., 2022). Participants in our cohort received vaccines 6-14 months after the initial infection. Consistent with previous reports (Marcotte et al., 2022), we found a trend towards higher levels of neutralization activity with increased timing between infection and vaccination in the 6-14 month period analyzed, which was significant in the case of Omicron. This suggests that intervals greater than 6 months between infection and vaccination might further improve the magnitude and breath of the neutralizing antibody response induced by vaccination.

Although SARS-CoV-2 poses a much higher risk of severe disease in elderly people (Bar-Haim et al., 2022; Bar-On et al., 2022; Vashishtha and Kumar 2022), in this study we observed the immune response in a cohort of young adults who experienced asymptomatic and pauci-symptomatic infection and are epidemiologically relevant as they could facilitate viral transmission to more vulnerable populations. Only a limited number of studies have been able to assess immune responses in this age group. Although younger adults represent a large number of infections and are important for the spread of disease, this group has been understudied owing to the difficulty in identifying asymptotic infections. A strength of this study is the high number of asymptomatic cases, which are usually difficult to assess. Additional strengths of this study include the use of samples from a well-characterized cohort, with precise information about the timing of the infection, course of infection, clinical profile, and timing and type of vaccination.

In summary, our study describes a series of immunological measurements demonstrating that vaccination significantly improves immune memory among previously infected young adults and that notably, this gain is comparable after asymptomatic or mildly symptomatic infection, including against Omicron.

### Limitations of the study

As this is a cross-sectional study, the timing of antigenic exposure between the groups is different. The infected and vaccinated group had a greater time between last known exposure to antigen and enrollment in this study when compared to participants who were infected and vaccinated. This difference may limit our ability to compare the dynamics of immune responses between infection alone and infection followed by vaccination on the same time scale. Notably, the time between the first positive PCR to enrollment in the unvaccinated group (254 ± 79 days) and the time between first PCR to the first dose of vaccination in the vaccinated group (251 ± 54 days) were similar (Figure 1), which suggested that our results on unvaccinated participants reflect a similar immunologic status for the vaccinated participants prior to receiving vaccination. In this study, we could not identify a group of “uninfected-vaccinated” participants, as participants that remained PCR negative and seronegative at the end of the CHARM study might have been exposed before sample collection at the CHARM 2.0 study. Thus, we were not able to compare the immune response in uninfected-vaccinated participants with that of infected-unvaccinated or infected-vaccinated participants. Other study limitations include a population mostly comprised of young adult males; additionally, all participants were previously infected with the Ancestral strain of SARS-CoV-2. As this was a cross-sectional study, data were not available from a few weeks after the time of 1^st^ PCR detection until the time of sample collection which could have led to additional acute exposure to SARS-CoV-2 or VOCs that were not observed as part of this study through PCR detection. Finally, our study was not designed to address booster doses in these young adults owing to the timing of the study. The beginning of 2020 was when booster doses were just begun to be distributed. Additional studies assessing booster doses and immunologic status against contemporary SARS-CoV-2 variants are needed.

## STAR★Methods

### Key resources table

**Table:** 

REAGENT or RESOURCE	SOURCE	IDENTIFIER
**Antibodies**		

Mouse anti-human CD8 BUV496 (clone RPA-T8)	BD Biosciences	Cat# 612942; RRID:AB_2870223
Mouse anti-human CD3 BUV805 (clone UCHT1)	BD Biosciences	Cat# 612895; RRID:AB_2870183
Mouse anti-human CD14 V500 (clone M5E2)	BD Biosciences	Cat# 561391; RRID:AB_10611856
Mouse anti-human CD19 V500 (clone HIB19)	BD Biosciences	Cat# 561121; RRID:AB_10562391
Mouse anti-human CD4 BV605 (clone RPA-T4)	BD Biosciences	Cat# 562658; RRID:AB_2744420
Mouse anti-human CD69 PE (clone FN50)	BD Biosciences	Cat# 555531; RRID:AB_395916
Mouse anti-human CD134 (OX40) PE-Cy7 (clone Ber-ACT35)	BioLegend	Cat# 350012; RRID:AB_10901161
Mouse anti-human CD137 APC (clone 4B4-1)	BioLegend	Cat# 309810; RRID:AB_830672
Mouse anti-human CD154 (CD40 Ligand) APC-ef780 (clone 24–31)	eBioscience (Thermo Fisher Scientific)	Cat# 47-1548-42; RRID:AB_1603203
Rat anti-human CXCR5 (CD185) BB700 (clone RF8B2)	BD Biosciences	Cat# 566469; RRID:AB_2869769
Mouse anti-human CD279 (PD-1) PE-Dazzle594 (clone EH12.2H7)	BioLegend	Cat# 329940; RRID:AB_2563659
Mouse anti-human CD19 BUV563 (clone SJ25C1)	BD Biosciences	Cat# 612916; RRID:AB_2870201
Mouse anti-human IgD Pacific Blue (clone IA6-2)	BioLegend	Cat# 348224; RRID:AB_2561597
Mouse anti-human CD20 BV510 (clone 2H7)	BioLegend	Cat# 302340; RRID:AB_2561941
Mouse anti-human IgM BV570 (clone MHM-88)	BioLegend	Cat# 314517; RRID:AB_10913816
Mouse anti-human CD27 BB515 (clone M-T271)	BD Biosciences	Cat# 564642; RRID:AB_2744354
Mouse anti-human IgA Vio Bright FITC (clone IS11-8E10)	Miltenyi Biotec	Cat# 130-113-480; RRID:AB_2734076
Mouse anti-human CD3 PerCP (clone SK7)	BioLegend	Cat# 344814; RRID:AB_10639948
Mouse anti-human CD14 PerCP (clone 63D3)	BioLegend	Cat# 367152; RRID:AB_2876693
Mouse anti-human CD16 PerCP (clone 3G8)	BioLegend	Cat# 302030; RRID:AB_940380
Mouse anti-human CD56 PerCP (clone 3G8)	BioLegend	Cat# 318342; RRID:AB_2561865
Mouse anti-human IgG PerCP/Cyanine5.5 (clone M1310G05)	BioLegend	Cat# 410710; RRID:AB_2565788
Mouse anti-human CD38 APC/Fire 810 (clone HIT2)	BioLegend	Cat# 303550; RRID:AB_2860784
Goat F(ab')2 Anti-Human IgG (Fab')2 (HRP)	Abcam	Cat# ab98535

**Biological samples**		

Human serum	CHARM 2.0 study	N/A
PBMC	CHARM 2.0 study	N/A
Human serum collected before July 2019 (negative controls for ELISA assays)	Biochemed	N/A

**Chemicals, peptides, and recombinant proteins**		

Brilliant Staining Buffer Plus	BD Biosciences	Cat# 566385
BD Horizon Brilliant Stain Buffer	BD Biosciences	Cat# 566349
Live/Dead Viability Dye eFluor506	Invitrogen (Thermo Fisher Scientific)	Cat# 65-0866-14
Live/Dead Fixable Blue Stain Kit	Thermo Fisher Scientific	Cat# L34962
Synthetic peptides	TC Peptide Lab	https://www.tcpeptide.com
Ancestral (WT) S SARS-CoV-2 Protein	AcroBiosystems	Cat# SPN-C82E9
Beta (B.1.351) S SARS-CoV-2 Protein	AcroBiosystems	Cat# SPN-C82E4
Delta (B.1.617.2) S SARS-CoV-2 Protein	AcroBiosystems	Cat# SPN-C82Ec
Omicron (B.1.1.529) S SARS-CoV-2 Protein	AcroBiosystems	Cat# SPN-C82Ee
Ancestral (WT) S1 D614G SARS-CoV-2 Protein	Sino Biological	Cat# 40591-V08H3
Delta (B.1.617.2) S1 SARS-CoV-2 Protein	Sino Biological	Cat# 40591-V08H23
Omicron (B.1.1.529) S1 SARS-CoV-2 Protein	Sino Biological	Cat# 40591-V08H41
Ancestral (WT) S Ectodomain trimer D614G SARS-CoV-2 Protein	Sino Biological	Cat# 40589-V08H8
Delta (B.1.617.2) S Ectodomain trimer SARS-CoV-2 Protein	Sino Biological	Cat# 40589-V08H10
Omicron S Ectodomain trimer SARS-CoV-2 Protein	Sino Biological	Cat# 40589-V08H26
Ancestral (WT) SARS-CoV-2 NTD SARS-CoV-2 Protein	AcroBiosystems	Cat# S1D-C52H6
Delta (B.1.617.2) NTD SARS-CoV-2 Protein	AcroBiosystems	Cat# S1D-C52Hh
Omicron (B.1.1.529) NTD SARS-CoV-2 Protein	AcroBiosystems	Cat# SPD-C522d
Brilliant Violet 711 Streptavidin	BioLegend	Cat# 405241
Brilliant Violet 421 Streptavidin	BioLegend	Cat# 405225
BD Horizon BUV737 Streptavidin	BD Biosciences	Cat# 612775
Streptavidin, Alexa Fluor 647 conjugate	Thermo Fisher Scientific	Cat# S21374
BD Horizon BUV615 Streptavidin	BioLegend	Cat# 613013
Streptavidin, (PE-Cy5.5)	Thermo Fisher Scientific	Cat# SA1018
o-phenylenediamine (OPD)	Sigma-Aldrich	Cat# P5412-100TAB
Phosphate-Citrate Buffer with Sodium Perborate for OPD	Sigma-Aldrich	P4922-100CAP
Hydrochloric acid, ACS reagent, ca. 37% solution in water	Fisher Scientific	Cat# AC423795000

**Experimental models: Cell lines**		

HEK293	ATCC	Cat# CRL-1573
HEK293t/17	ATCC	Cat# CRL-11268
HEK293-ACE2	NMRC	Produced in house

**Oligonucleotides**		

Spike genes for generation of pseudoviruses	GenScript	N/A

**Recombinant DNA**		

pcDNA3.1 + expression vectors	Thermo Fisher Scientific	Cat# V79020
Lentivirus derived reporter and packaging plasmids	BEI resources	NR-53817 (Crawford et al., (2020)
ACE2 expression vector for transfection of HEK293T	GeneCopoeia	Cat# U1285

**Software and algorithms**		

GraphPad Prism 9	GraphPad	https://www.graphpad.com/; RRID:SCR_002798
FlowJo 10	FlowJo	https://www.flowjo.com/; RRID:SCR_008520
IEDB	(Grifoni et al., 2020)	https://www.iedb.org; RRID:SCR_006604
R version 4.0.4	The R Project for Statistical Computing	https://www.r-project.org/
RStudio version 1.3.1093	RStudio	https://www.rstudio.com/

### Resource availability

#### Lead contact

Further information and requests for resources and reagents should be directed to and will be fulfilled by the lead contact author, Dr. Andrew G. Letizia (Andrew.g.letizia.mil@mail.mil)

#### Materials availability

This study did not generate new unique reagents.

### Experimental model and subject details

#### Human subjects

The initial COVID-19 Health Action Response for Marines (CHARM) study was a longitudinal, prospective study that enrolled 3,472 U.S. Marine Corps (USMC) recruits at Marine Corps Recruiting Depot – Parris Island, between May and November 2020, to investigate the epidemiology and the immune response to SARS-CoV-2. The approximately 2,900 recruits who graduated from recruit training and became active-duty Marines in the original CHARM study were eligible for a follow-up, repeated cross-sectional study called CHARM 2.0 which was designed to assess the long-term effects of infection, including various aspects of the adaptive immune response. Between February and November 2021, the field researchers visited 11 DoD facilities in 4 states enrolling Marines who were garrisoned and not performing field operations or deployed. Consenting Marines completed an extensive clinical questionnaire to record clinical information and vaccine history and provided blood and nares (nasal) samples at each visit. In this sub-study, we emphasized variants of greatest concern for immune escape on groups of participants infected with SARS-CoV-2 during the initial CHARM study and then who were or not fully vaccinated at least 28 days after receiving a second dose of a mRNA vaccine. The time between the initial infection and the follow-up visits in CHARM 2.0 varied based upon when the Marines attended recruit training and became infected during CHARM, and when the field researchers traveled to where the Marines were stationed during CHARM 2.0. Sample selection for the present study is described in Figure 1.

The average age of the sample population at enrollment in the CHARM study was 19.1 ± 1.9 years, 13.3% identified as female. Since this was a homogeneous population in regards to sex and age and was not powered to address associations of age and gender on outcomes, these analyses were not performed.

The study protocols were approved by the Naval Medical Research Center Institutional Review Board (protocol numbers NMRC.2020.0006 and NMRC.2021.0004) in compliance with all applicable federal regulations governing the protection of human subjects. All participants provided written informed consent for participation.

Blood was collected using serum separator tubes (SST) for serum and heparin tubes (BD Vacutainer) for peripheral blood mononuclear cells (PBMCs). Serum Separator Tubes (SST) were centrifuged to obtain serum (1500 × g for 10 min). PBMCs were isolated using Accuspin tubes (Sigma-Aldrich) and Histopaque 1077 (Sigma-Aldrich), following manufacture’s recommendations.

### Method details

#### Pseudovirus neutralization assay

Pseudovirus (PV) was produced using SARS-CoV-2 Spike (S) gene sequences obtained from the GSAID database (www.gsaid.org). Wuhan-Hu-1 strain (termed Ancestral hereafter) with the D614G mutation, Delta (B.1.617.2) and Omicron (B.1.1.529) S gene sequences were codon optimized for human expression, with 19 amino acids removed from the C-terminus. S genes were synthesized by GenScript (Piscataway, NJ) and cloned in to pcDNA3.1 + expression vectors. Lentivirus derived reporter and packaging plasmids have been previously described by (Crawford et al., 2020), and generously made available through BEI resources (NIAID, NIH). PV was produced in HEK293T/17 cells obtained from ATCC (Manassas, VA) and transfected with S, ZsGreen reporter and packaging plasmids using Lipofectamine 3000 reagent (Thermo Fisher Scientific, MA). HEK293-ACE2 expressing cell lines were developed using HEK293 cells obtained from ATCC, transfected with an ACE2 expression vector (GeneCopoeia; Rockville, MD). Stable transfectants were selected using G418 (Invivogen), and individual sub-clones were selected based on maximal ACE2 expression. HEK293-ACE2 cells were cultured in DMEM (Gibco) containing 10% FBS (GE) and 1× Penicillin/Streptomycin (Quality Biological) and supplemented with G418.

Neutralization assays were performed using 96-well flat bottom plates (Corning). Serum was heat inactivated at 56°C for one hour prior to use and sample serum along with controls were then diluted 1:20 in DMEM containing 10% FBS, followed by 1:2 serial dilutions. PV was then added to the serum dilutions and the mixture was incubated at 37°C, 5% CO_2_ for 30 min to allow for antibody binding. HEK293-ACE2 cells were then added and plates retuned to the 37°C incubator for 48 h, after which time, cells were treated with trypsin (Quality Biological) and re-suspended in 1X PBS (Gibco) supplemented with 10% FBS. Plates were read using a FACS Canto II equipped with an HTS running BD FACSDiva software (Version 8.0.1). Data was analyzed by plotting percent positive cells as a function of log dilution using Prism GraphPad software (Version 8.3.1). Nonlinear regression analysis was then used to calculate the 50% neutralizing dose (ND_50_). If a sample did not show neutralization at the lowest serum dilution (1:20), this sample was scored as negative and assigned a number 20 for data presentation and analysis.

#### IgG antibody binding assays

Serum IgG antibody binding to RBD, S1, NTD and S trimer of the Ancestral, Delta and Omicron variants were quantified using an enzyme-linked immunosorbent assay (ELISA). Immulon 4 HBX 384-well plates (Thermo Fisher Scientific, Waltham, MA, UA), were coated overnight at 4°C with 2 μg/mL of the following recombinant His-tagged proteins (detailed information in Table S1): SARS-CoV-2 RBD (Sino Biological); SARS-CoV-2 S1 D614G protein (Sino Biological); SARS-CoV-2 S Ectodomain trimer D614G (Sino Biological); SARS-CoV-2 NTD (Acrobiosystems); Delta (B.1.617.2) RBD Protein (Sino Biological); Delta S1 Protein (Sino Biological); Delta S Ectodomain trimer (Sino Biological); Delta NTD (Acrobiosystems); Omicron (B.1.1.529) RBD Protein (Sino Biological); Omicron S1 Protein (Sino Biological); Omicron S Ectodomain trimer (Sino Biological); Omicron NTD (Acrobiosystems). Plates were washed with 0.1% Tween-20 PBS buffer using an automated ELISA plate washer (AquaMax 4000, Molecular Devices, San Jose, CA, USA), and blocked for 1 h at room temperature with 3% milk in PBS-T. Blocking solution was removed, and six serial dilutions of serum (3-fold dilutions starting at 1:50, prepared in 1% milk PBS-T) were dispensed in the wells. At least two positive controls (sera with known IgG presence), eight negative controls (sera collected before July, 2019, Biochemed, VA, USA), and four blanks (no serum) were included in every assay. Plates were incubated for 2 h at room temperature and washed. Next, peroxidase conjugated goat F(ab')2 Anti-Human IgG (Abcam, Cambridge, UK) was added at 1:8000 dilution (determined after optimization for antibody lot) in 1% milk PBS-T, and plates were incubated for 1 h at room temperature. Plates were washed six times, developed using o-phenylenediamine, and the reaction was stopped after 10 min with 3M HCl. Optical density (OD) at 492 nm was measured using a microplate reader (SpectraMax M2, Molecular Devices). The Hudson SOLO™ automated pipettor was used for all pipetting steps except for the coating of plates, washing steps and preparation of dilutions. Each dilution was considered positive when its OD 492 nm value was higher than the average of the negative controls plus 3 times their standard deviation (SD) at the correspondent dilution, and higher than OD 492 nm of 0.15. Samples were considered positive for RBD, S1, NTD or S trimer reactive IgG when positive results were obtained for at least two consecutive dilutions (endpoint titer of 150 or higher). Area under the curve (AUC) values were calculated using the six dilutions assayed for RBD, S1, NTD or S IgG antibodies.

#### Flow cytometry-based B cell assays

Quantification of antigen-specific B cells by flow cytometry was performed using B cell probes consisting of SARS-CoV-2 S proteins conjugated with fluorescent streptavidin, as previously described (Tarke et al., 2022). Recombinant proteins used in this study are described in Table S1. To enhance specificity, the identification of Ancestral protein was performed using two fluorochromes, prior to gating on variant B cells (Figure S1). For that, biotinylated Ancestral SARS-CoV-2 S was incubated with streptavidin in either BV711 (BioLegend) or BV421 (BioLegend) at a 20:1 ratio (∼6:1 molar ratio) for 1 h at 4°C. The streptavidin-fluorochrome conjugates used to tetramerize the SARS-CoV-2 variant proteins are listed as follows: Omicron S BUV737 (BD Biosciences), Delta S, Alexa Fluor 647, (Thermo Fischer Scientific), Beta S (B.1.351) BUV615, (BD Biosciences). Streptavidin PE-Cy5.5 (Thermo Fisher Scientific) was used as a decoy probe to minimize background by eliminating SARS-CoV-2 nonspecific streptavidin-binding B cells. Average of 1.5 to 4 million PBMCs were placed in U-bottom 96 well plates and stained with a solution consisting in 5 μg of biotin (Avidity, catalog no. Bir500A) to avoid cross reactivity among probes, 20 ng of decoy probe, 416 ng of S and 20.1 ng of RBD per sample, diluted in Brilliant Buffer (BD Biosciences) and incubated for 1 h at 4°C, protected from light. After washing with PBS, cells from both S and RBD panels were incubated with surface antibodies diluted in Brilliant Buffer, for 30 at 4°C, protected from light. The viability staining was performed using Live/Dead Fixable Blue Stain Kit (Thermo Fisher) diluted 1:200 in PBS and incubated at 4°C for 30 min. The acquisition was performed on Cytek Aurora and analyses were made using Flow Jo v. 10.7.1 (BD Biosciences). The frequency of Variants-specific memory B cells was expressed as a percentage of Ancestral S or RBD memory B cells (Singlets, Lymphocytes, Live, CD3– CD14– CD16– CD56–CD19+ CD20^+^ CD38int/–, IgD– and/or CD27^+^ S or RBD BV711+, S or RBD BV421+). PBMCs from a known positive control (COVID-19 convalescent subject) and an unexposed subject were included to ensure consistent sensitivity and specificity of the assay. The limit of detection was calculated as median + 2xSD of [1/(number of total B cells recorded)].

#### Peptide synthesis and megapool preparation for Activation Induced Marker (AIM) assays

All peptides were synthesized as crude material (TC Peptide Lab, San Diego, CA) and individually resuspended in dimethyl sulfoxide (DMSO) at concentrations of 10–20 mg/mL. To prepare S Megapools (MP) (Table S2), sets of 15-mer peptides overlapping by 10 amino acids were synthetized in order to span the whole SARS-CoV-2 S protein corresponding to the ancestral Wuhan sequence, as well as a selection of the SARS-CoV-2 variants [Beta (L18F, D80A, D215G, D215H, L241del, L242del, A243del, K417N, E484K, N501Y, D614G, A701V), Delta (T19R, L452R, T478K, D614G, P681R, D950N), and Omicron (A67V, H69del, V70del, T95I, G142D, V143del, Y144del, Y145del, W152del, N211del, L212I, ins214EPE, G339D, S371L, S373P, S375F, K417N, N440K, G446S, S477N, T478K, E484A, Q493R, G496S, Q498R, N501Y, T547K, D614G, H655Y, N679K, P681H, N764K, D796Y, N856K, Q954H, N969K, L981F)]. The MP for each variant was made by pooling aliquots of the corresponding individual peptides which were further sequentially lyophilized. The resulting lyophilized MP was resuspended in DMSO at 1 mg/mL as previously described (Tarke et al., 2021; Tarke et al., 2022).

#### AIM T cell assays

Activation Induced Marker (AIM) assays were described previously (Tarke et al., 2021; Tarke et al., 2022). PBMCs were cultured in the presence of SARS-CoV-2-specific (Ancestral or variant) MPs [1 μg/mL] in 96-well U-bottom plates at a concentration of 1 × 10^6^ PBMCs per well. The negative control was an equimolar of DMSO that contained antigens and added to the cells in triplicate wells. For a positive control, phytohemagglutinin (PHA, Roche, 1 μg/mL) was used to stimulate cells. CXCR5-BB700 (BD, 1:100) and CD40 (Miltenyi, 1:200) were added in culture to all conditions.

After incubation for 24 h at 37°C in 5% CO_2_, cells were washed and stained for AIM and circulating T follicular helper (cT_FH_) markers on their surface for 30 min at 4°C in the dark. The staining cocktail included antibodies for CD3-BUV805 (BD, 1:50), CD8-BUV496 (BD, 1:50), CD4-BV605 (BD, 1:100), CD14-V500 (BD, 1:50), and CD19-V500 (BD, 1:50) as well as the viability dye eFluor506 (Invitrogen, 1:400) (Table S3). Activation markers were added for OX40-PE-Cy7 (Biolegend, 1:50), CD40L-APC-eFluor780 (eBioscience, 3:100), CD69-PE (BD, 1:10), and CD137-APC (Biolegend, 1:25). Additionally, the antibodies for CXCR5-BB700 (BD, 1:50) and PD-1-PE-Dazzle594 (Biolegend, 1:25) were added to allow cT_FH_ detection. After staining, cells were then acquired directly on a ZE5 5-laser cell analyzer (Bio-Rad laboratories) and analyzed with FlowJo software (Tree Star Inc.).

The gating strategy for AIM cells was drawn relative to the negative and positive controls for each donor. Specifically, lymphocytes were first gated and then single cells were selected to exclude doublets. T cells were gated based on CD3 positivity in combination with negativity for a Dump channel that included (in the same colors) CD14, CD19 and Live/Dead viability dye. The CD3^+^CD4^+^ and CD3^+^CD8^+^ were further gated based on OX40+CD137+, OX40 + CD40L + or CD69^+^CD137+ AIM markers, respectively. cT_FH_ + cells were selected based on the CXCR5+ expression of AIM+ CD4^+^ T cells. To analyze the resulting data, the background was first removed from the data by subtracting the average of the percent of AIM + cells stimulated with DMSO in triplicate. The Stimulation Index (SI) was calculated by dividing the % of AIM + cells after SARS-CoV-2 stimulation with the average % of AIM + cells in the negative DMSO control.

The limit of sensitivity (LOS) was 0.03% or 0.04% AIM+ CD4^+^ or CD8^+^ cells, based on the median twofold standard deviation of T cell reactivity in negative DMSO controls.

A response was considered positive when greater than the LOS and the SI was greater than 2, after background subtraction.

### Quantification and statistical analysis

FlowJo 10, GraphPad Prism 8.4, R (version 4.0.4), and RStudio (version 1.3.1093) were used for data and statistical analyses, unless otherwise stated (Table S4). Pairwise comparisons of immune parameters were performed with the Mann-Whitney Test, and frequencies were compared using the Fisher Exact Test. Comparison of T cell responses specific for the different VOCs were done using the Kruskal-Wallis test. Statistical comparisons of neutralization (ND_50_ values) and antibody binding AUC values were done using Log10 transformed data. Simple linear regression models were used to evaluate the effect of time between infection and vaccination on the antibody immune response to vaccines in the infected-vaccinated group. A significant effect was considered when the slope of each model was different than 0 (alpha = 0.05, ANOVA). The details for the statistics of each experiment are provided in the respective figure legends as well as in the respective methods.

## Data Availability

All data reported in this paper and deemed non-sensitive by the US Navy will be shared by the lead contact upon request Any additional information required to reanalyze the data reported in this paper is available from the lead contact upon request and with the approval of the US Navy This paper does not report original code.

## Supplemental information

Document S1. Tables S1, S3, S4, and Figures S1–S6

Table S2. List of peptides contained in each spike variant pool related to STAR Mathods
