# Supplementary material for: Asymptomatic or symptomatic SARS-CoV-2 infection plus vaccination confers increased adaptive immunity to variants of concern
Source: iScience. 2022 Sep 23;25(10):105202. doi: 10.1016/j.isci.2022.105202 (PMC9502440; doi:10.1016/j.isci.2022.105202)
Supplement: Document S1. Tables S1, S3, S4, and Figures S1–S6 [file mmc1.pdf]

iScience, Volume *25*

Supplemental information

Asymptomatic or symptomatic SARS-CoV-2 infection plus vaccination confers increased adaptive immunity to variants of concern

Peifang Sun, Irene Ramos, Camila H. Coelho, Alba Grifoni, Corey A. Balinsky, Sindhu Vangeti, Alison Tarke, Nathaniel I. Bloom, Vihasi Jani, Silvia J. Jakubski, David A. Boulifard, Elizabeth Cooper, Carl W. Goforth, Jan Marayag, Amethyst Marrone, Edgar Nunez, Lindsey White, Chad K. Porter, Victor A. Sugiharto, Megan Schilling, Avinash S. Mahajan, Charmagne Beckett, Alessandro Sette, Stuart C. Sealfon, Shane Crotty, and Andrew G. Letizia

Supplemental Material

Table S1. Chemicals, peptides, and recombinant proteins related to STAR Methods

Reagent name	Manufacturer	Ordering information
Brilliant Staining Buffer Plus	BD Biosciences	Cat# 566385
BD Horizon Brilliant Stain Buffer	BD Biosciences	Cat# 566349
Live/Dead Viability Dye eFluor506	Invitrogen (Thermo Fisher Scientific)	Cat# 65-0866-14
Live/Dead Fixable Blue Stain Kit	Thermo Fisher Scientific	Cat# L34962
Synthetic peptides	TC Peptide Lab	https://www.tcpeptide.com
Ancestral (WT) S Protein	AcroBiosystems	Cat# SPN-C82E9
Beta (B.1.351) S Protein	AcroBiosystems	Cat# SPN-C82E4
Delta (B.1.617.2) S Protein	AcroBiosystems	Cat# SPN-C82Ec
Omicron (B.1.1.529) S Protein	AcroBiosystems	Cat# SPN-C82Ee
SARS-CoV-2 RBD	Sino Biological	Cat# 40592-V08H
Delta (B.1.617.2) RBD Protein	Sino Biological	Cat# 40592-V08H90
Omicron (B.1.1.529) RBD Protein	Sino Biological	Cat# 40592-V08H121
SARS-CoV-2 S1 D614G	Sino Biological	Cat# 40591-V08H3
Delta S1 Protein	Sino Biological	Cat# 40591-V08H23
Omicron S1 Protein	Sino Biological	Cat# 40591-V08H41
SARS-CoV-2 S Ectodomain trimer D614G	Sino Biological	Cat# 40589-V08H8
Delta S Ectodomain trimer	Sino Biological	Cat# 40589-V08H10
Omicron S Ectodomain trimer	Sino Biological	Cat# 40589-V08H26
SARS-CoV-2 NTD	AcroBiosystems	Cat# S1D-C52H6
Delta NTD	AcroBiosystems	Cat# S1D-C52Hh
Omicron NTD	AcroBiosystems	Cat# SPD-C522d

Table S3. T cell assay reagents related to STAR Methods

REAGENT or RESOURCE	SOURCE	IDENTIFIER
Mouse anti-human CD8 BUV496 (clone RPA-T8)	BD Biosciences	Cat# 612942; RRID:AB_2870223
Mouse anti-human CD3 BUV805 (clone UCHT1)	BD Biosciences	Cat# 612895; RRID:AB_2870183
Mouse anti-human CD14 V500 (clone M5E2)	BD Biosciences	Cat# 561391; RRID:AB_10611856
Mouse anti-human CD19 V500 (clone HIB19)	BD Biosciences	Cat# 561121; RRID:AB_10562391
Mouse anti-human CD4 BV605 (clone RPA-T4)	BD Biosciences	Cat# 562658; RRID:AB_2744420
Mouse anti-human CD69 PE (clone FN50)	BD Biosciences	Cat# 555531; RRID:AB_395916
Mouse anti-human CD134 (OX40) PE-Cy7 (clone Ber-ACT35)	BioLegend	Cat# 350012; RRID:AB_10901161
Mouse anti-human CD137 APC (clone 4B4-1)	BioLegend	Cat# 309810; RRID:AB_830672
Mouse anti-human CD154 (CD40 Ligand) APC-ef780 (clone 24-31)	eBioscience (Thermo Fisher Scientific)	Cat# 47-1548-42; RRID:AB_1603203
Rat anti-human CXCR5 (CD185) BB700 (clone RF8B2)	BD Biosciences	Cat# 566469; RRID:AB_2869769
Mouse anti-human CD279 (PD-1) PE-Dazzle594 (clone EH12.2H7)	BioLegend	Cat# 329940; RRID:AB_2563659
Mouse anti-human CD19 BUV563 (clone SJ25C1)	BD Biosciences	Cat# 612916; RRID:AB_2870201
Mouse anti-human IgD Pacific Blue (clone IA6-2)	BioLegend	Cat# 348224; RRID:AB_2561597
Mouse anti-human CD20 BV510 (clone 2H7)	BioLegend	Cat# 302340; RRID:AB_2561941
Mouse anti-human IgM BV570 (clone MHM-88)	BioLegend	Cat# 314517; RRID:AB_10913816
Mouse anti-human CD27 BB515 (clone M-T271)	BD Biosciences	Cat# 564642; RRID:AB_2744354
Mouse anti-human IgA Vio Bright FITC (clone IS11-8E10)	Miltenyi Biotec	Cat# 130-113-480; RRID:AB_2734076
Mouse anti-human CD3 PerCP (clone SK7)	BioLegend	Cat# 344814; RRID:AB_10639948
Mouse anti-human CD14 PerCP (clone 63D3)	BioLegend	Cat# 367152; RRID:AB_2876693

Mouse anti-human CD16 PerCP (clone 3G8)	BioLegend	Cat# 302030; RRID:AB_940380
Mouse anti-human CD56 PerCP (clone 3G8)	BioLegend	Cat# 318342; RRID:AB_2561865
Mouse anti-human IgG PerCP/Cyanine5.5 (clone M1310G05)	BioLegend	Cat# 410710; RRID:AB_2565788
Mouse anti-human CD38 APC/Fire 810 (clone HIT2)	BioLegend	Cat# 303550; RRID:AB_2860784
Brilliant Violet 711 Streptavidin	BioLegend	Cat# 405241
Brilliant Violet 421 Streptavidin	BioLegend	Cat# 405225
BD Horizon BUV737 Streptavidin	BD Biosciences	Cat# 612775
Streptavidin, Alexa Fluor 647 conjugate	Thermo Fisher Scientific	Cat# S21374
BD Horizon BUV615 Streptavidin	BioLegend	Cat# 613013
Streptavidin, (PE-Cy5.5)	Thermo Fisher Scientific	Cat# SA1018

Table S4. Software and algorithms used for data analysis Related to STAR Methods

GraphPad Prism 9	GraphPad	https://www.graphpad.com/; RRID:SCR_002798
FlowJo 10	FlowJo	https://www.flowjo.com/; RRID:SCR_008520
IEDB	Grifoni et al., 2020a	https://www.iedb.org; RRID:SCR_006604
R version 4.0.4		https://www.r-project.org/
RStudio version 1.3.1093		https://www.rstudio.com/

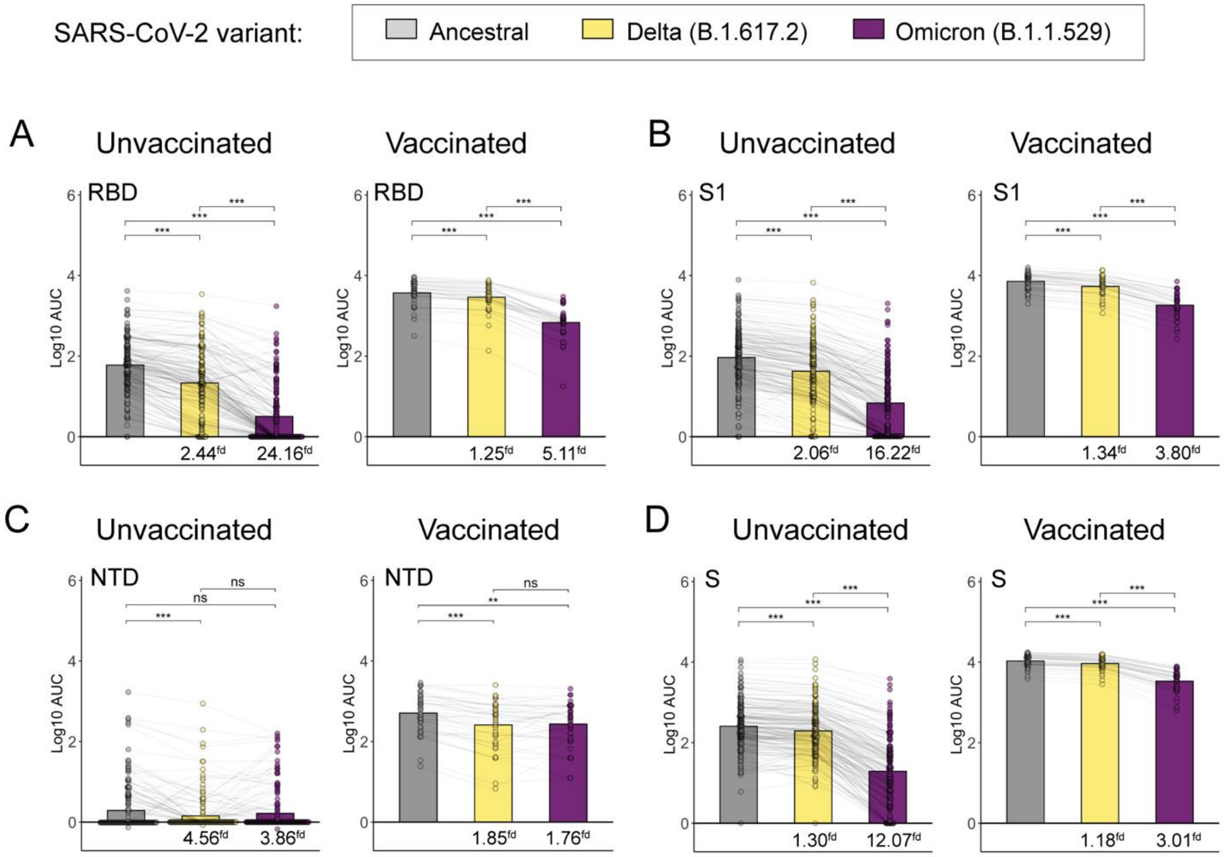

Figure S1. IgG binding to SARS-CoV-2 regions of S (A-C) or S trimers (D) of the Ancestral, Delta or Omicron variants in unvaccinated and vaccinated participants, related to Figure 3. Median fold-change decrease (^{fd}) as compared to the Ancestral strain is indicated under Delta and Omicron bars and was calculated considering only samples with positive IgG titers (≥150) against the Ancestral strain. AUC were compared using the Mann Whitney U test with Bonferroni correction for multiple comparisons. *** $p<0.001$; ** $p<0.01$; *$p<0.05$; $^{ns}p\geq0.05$.

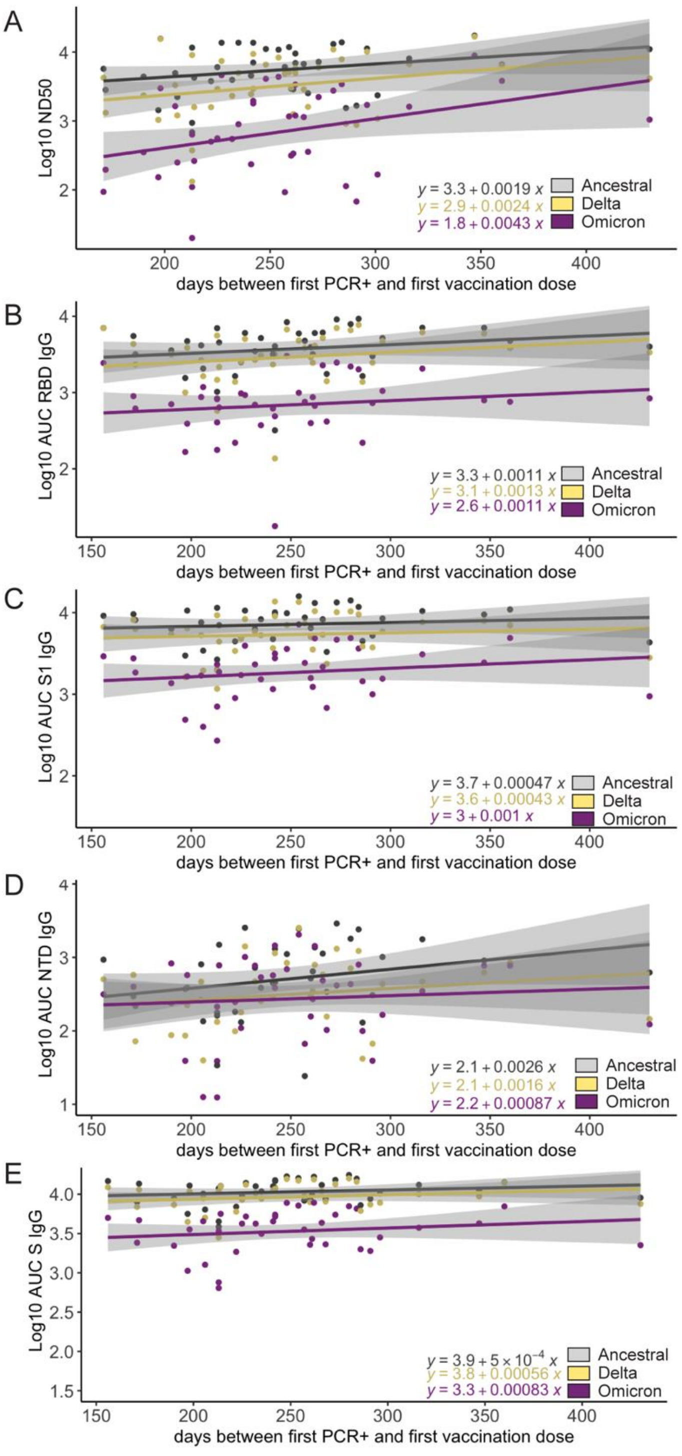

Figure S2. Neutralizing activity (A) and RBD IgG binding AUC values (B-E) in vaccinated participants with respect of timing between infection and first vaccine dose, related to Figures 2 and 3. Linear regression models are shown and were used to evaluate the effect of time between infection and vaccination on the antibody response. Neutralization: p-values = 0.085, 0.079 and 0.026 for Ancestral, Delta and Omicron, respectively (A); RBD binding: p-values = 0.223, 0.220 and 0.376 for Ancestral, Delta and Omicron, respectively (B); S1 binding: p-values = 0.482, 0.585 and 0.285 for Ancestral, Delta and Omicron, respectively (C); NTD binding: p-values = 0.082, 0.284 and 0.604 for

Ancestral, Delta and Omicron, respectively (D); S binding: p-values = 0.308, 0.297, 0.318 for Ancestral, Delta and Omicron, respectively (E).

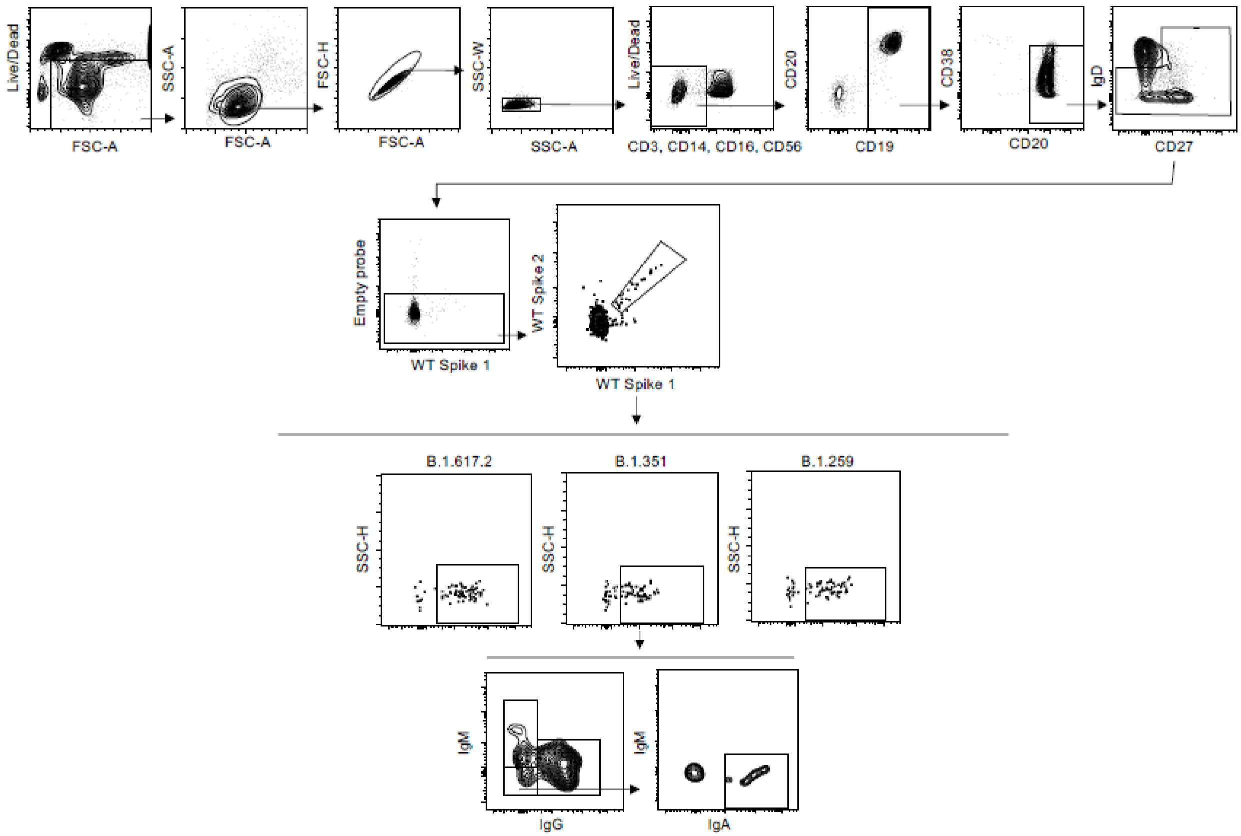

Figure S3. Gating strategy to identify variant-specific S memory B cells, related to Figure 4 and to STAR Methods.

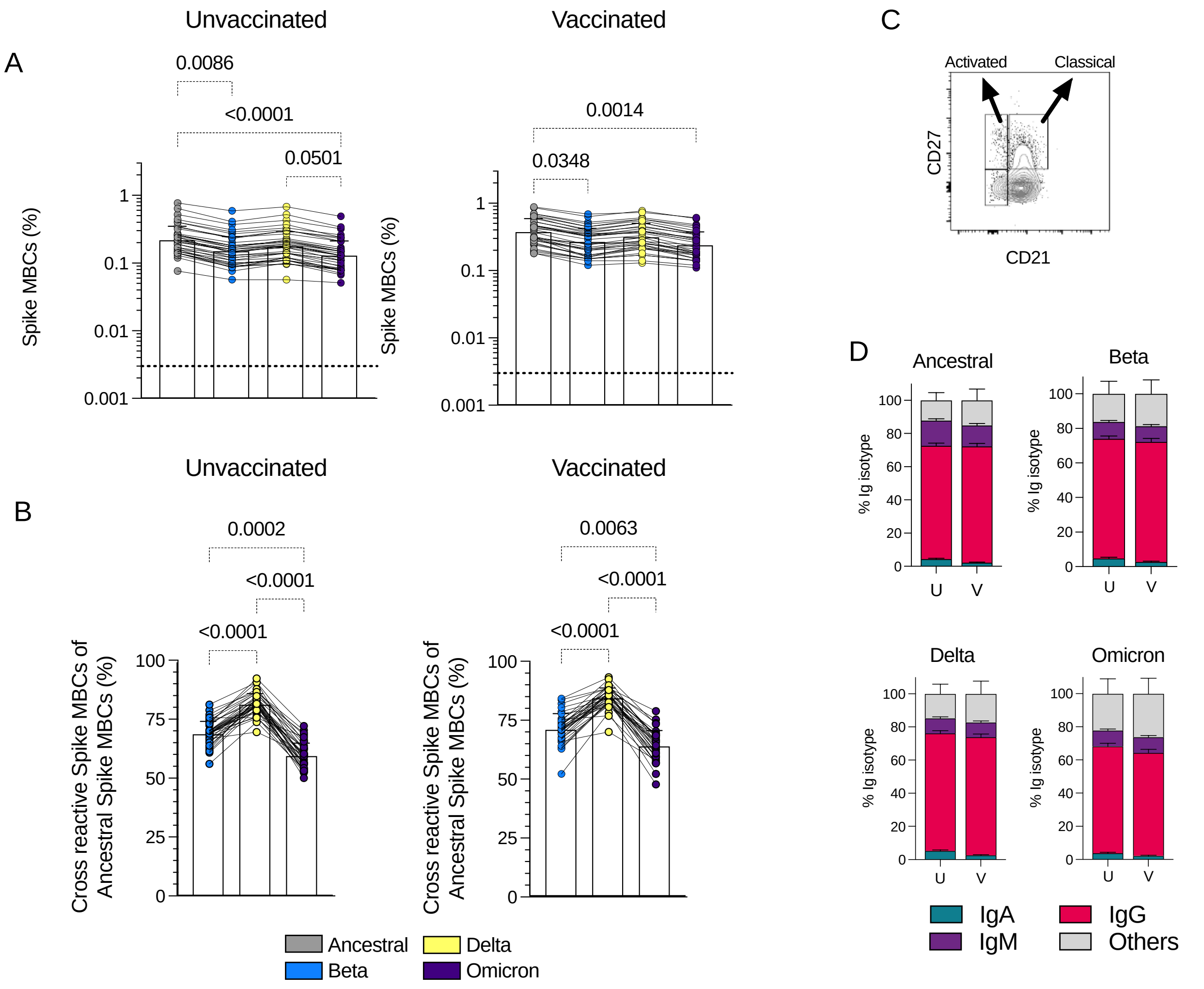

Figure S4. Variant-specific memory B cell response, related to Figure 4. (A) Frequency of S-specific memory B cells (MBCs) for each SARS-CoV-2 variant compared to Ancestral in unvaccinated and vaccinated subjects. (B) Frequency of cross-reactive S MBCs. (C) Flow cytometry gating for selection of activated and classical S-specific MBCs. (D) Isotype distribution in variant-specific S memory B cells. A-B were analyzed using Kruskal-Wallis test. In D, antibody isotypes compared among variants were analyzed using the Kruskal-Wallis test, and comparison between isotypes frequencies within a variant was performed using Fisher Exact test.

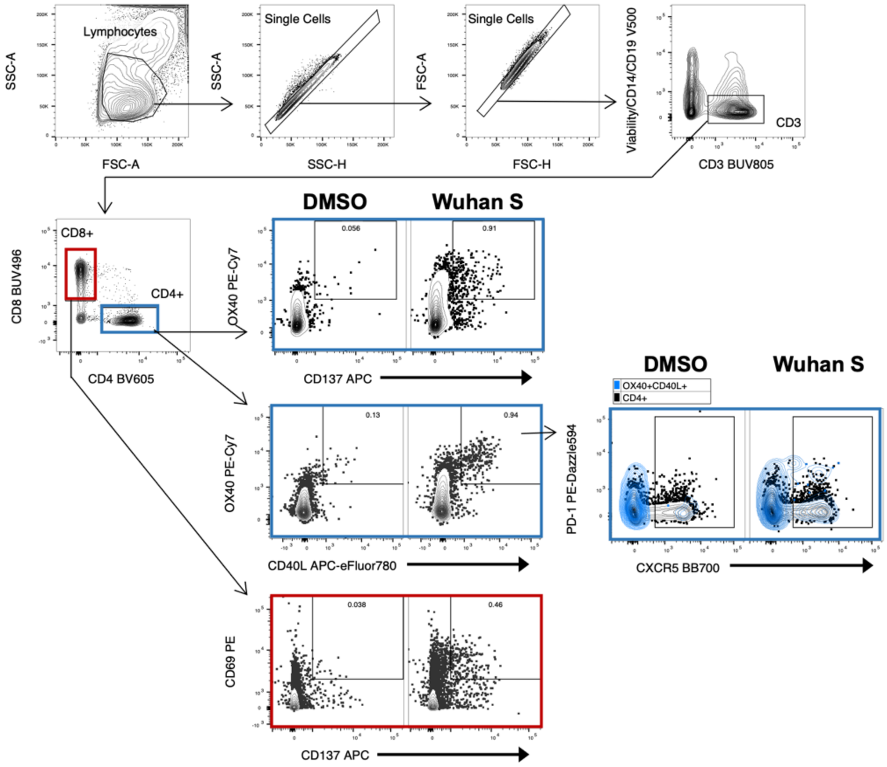

Figure S5. Gating strategy for flow cytometry-based AIM assay to assess SARS-CoV-2-specific T cells, related to Figure 5 and STAR Methods. A representative gating strategy is shown for the AIM T cell assay for a vaccinated and asymptomatic COVID-19 convalescent participants. S-specific AIM+ CD4+ T cells are measured by OX40+CD137+ and cT_{FH} are measured by CXCR5-expressing OX40+CD40L+ CD4+ T cells. S AIM+ CD8+ T cells are defined as double positive for CD69 and CD137. Representative graphs for the AIM and cT_{FH} markers are shown for DMSO and Wuhan S.

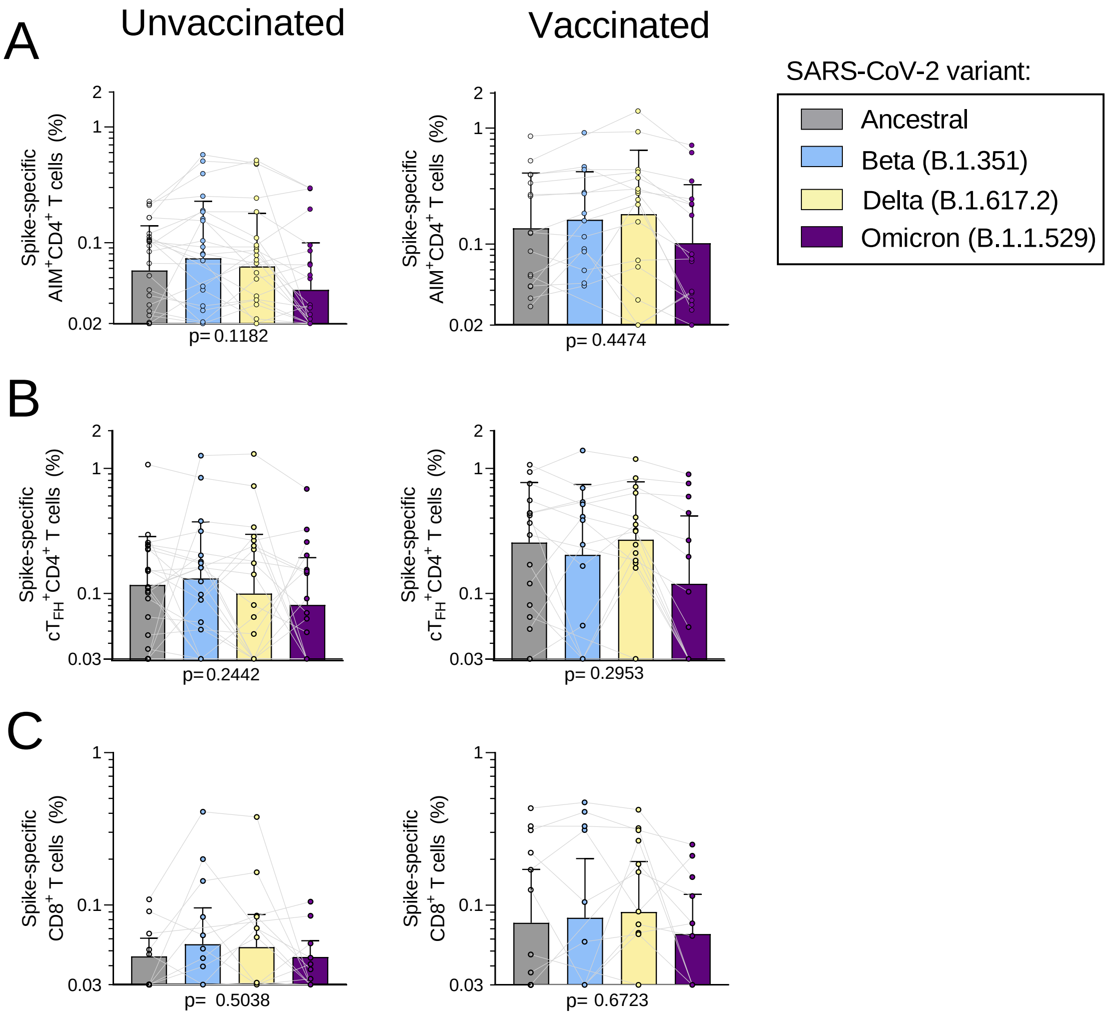

Figure S6. S-specific T cell response in unvaccinated and vaccinated individuals, related to Figure 5. Naturally infected Unvaccinated (left) and Vaccinated (right) samples are analyzed in terms of magnitude of response as percentage of S-specific cells for AIM^{+} ($OX40^{+}CD137^{+}$) $CD4^{+}$ T cells (**A**), AIM^{+} ($OX40^{+}CD40L^{+}$) cT_{fh} cells (**B**) and AIM^{+} ($CD69^{+}CD137^{+}$) $CD8^{+}$ T cells (**C**). In each cohort ancestral (grey), Beta (blue), Delta (yellow) and Omicron (purple) variants are compared by Kruskal-Wallis test.
